# Lysophosphatidic acid supports the development of vitrified ovarian follicles by decreasing the incidence of cell death: An experimental study

**DOI:** 10.18502/ijrm.v20i4.10899

**Published:** 2022-05-23

**Authors:** Neda Abedpour, Nassim Ghorbanmehr, Mojdeh Salehnia

**Affiliations:** ^1^Anatomy Department, Faculty of Medical Sciences, Tarbiat Modares University, Tehran, Iran.; ^2^Anatomy Department, Faculty of Medicine, Urmia University of Medical Sciences, Urmia, Iran.; ^3^Biotechnology Department, Faculty of Biological Sciences, Alzahra University, Tehran, Iran.

**Keywords:** Cell death, In vitro oocyte maturation, Lysophosphatidic acid, Lysophosphatidic acid receptors, Ovarian follicles, Vitrification.

## Abstract

**Background:**

Lysophosphatidic acid (LPA) contributes to follicular activation, oocyte maturation, in vitro fertilization, and embryo implantation.

**Objective:**

This study was designed to evaluate the effects of LPA to improve the development of isolated follicles derived from whole mouse cultured vitrified ovaries.

**Materials and Methods:**

In this experimental study, first, the 1-wk-old mouse ovaries in the non-vitrified and vitrified groups were cultured in the presence of 20 µM of LPA for 1 wk. Then, their isolated preantral follicles were cultured individually for 12 days in the presence or absence of 40 µM of LPA. The following evaluations were done for the cultured follicles: a viability test using Calcein AM staining, flow cytometry using annexin V/Pi, and analysis of the expression of genes by real-time reverse transcription polymerase chain reaction. The maturation rates of the oocytes were compared among groups and some of the released metaphase II oocytes were subjected to in vitro fertilization.

**Results:**

In all LPA treated groups, the rates of survival and follicular development were higher, and the incidence of cell death and expression of pro-apoptotic genes were lower, than in the non-LPA supplemented groups (p = 0.035). There was no significant difference between the vitrified and non-vitrified groups regarding follicular or oocyte development, but the expression of *Bad* and *LPA* receptors genes was significantly altered in the vitrified LPA supplemented group in comparison with the non-vitrified LPA supplemented group (p = 0.028).

**Conclusion:**

LPA improved the survival and developmental potential of the isolated follicles. Despite some alterations in the expression of apoptosis-related genes in the vitrified ovaries, LPA had positive effects on the survival and development of these follicles.

## 1. Introduction

The cryopreservation and in vitro culture of ovaries and isolated preantral follicles have been introduced as an alternative approach for fertility preservation. Several factors such as oxidative stress, hypoxia, and temperature alterations may influence the survival and development of ovarian follicles during in vitro growth and many attempts have focused on improving these procedures (1-3). Moreover, in the literature, it has been shown that cell death may be involved in follicular damage and degeneration following ovarian cryopreservation and in vitro culture (2, 3-7). Some advances have been achieved in the optimization of in vitro culture of follicles by using certain growth factors, antioxidants, and anti-apoptotic factors (3, 4, 8, 9).

Lysophosphatidic acid (LPA) is a natural transmembrane lysophospholipid with 430-480 Da molecular weight, and it has growth factor-like and hormone-like activities (10). LPA contributes to the regulation of female reproductive functions via autocrine and paracrine manners such as follicular activation, oocyte nuclear and cytoplasmic maturation, in vitro fertilization, and embryo implantation (10-12). LPA is present in human follicular fluid and it is produced by various cell types such as the cumulus and theca cells (13, 14). LPA carries out its biological functions through at least 6 high-affinity, transmembrane G-protein-coupled receptors (LPAR1-6) that are controlled by different genes in human, bovine, ovine, porcine, and rodent species (10, 15-19). LPARs have been found in reproductive tissues including the uterus and ovary (20), and progesterone and estradiol regulate LPAR expression (21, 22).

Several reports have revealed the beneficial effects of LPA supplementation with a range of concentrations between 10-30 µM/L in culture media to improve oocyte maturation and fertilization and subsequent embryo development in bovine, mouse, and pig species (23-28). Recently, in our previous studies, we showed that 20 µM of LPA had positive effects on cell survival and function of whole mouse ovaries during the cultivation period (29, 30). It was demonstrated that LPA, as a survival factor, enhanced the follicular development after transplantation of mouse ovaries by changing the pattern of the anti- and pro-apoptotic genes in association with miR-22 expression (31). Also, in another study revealed that the addition of LPA to culture media of human ovarian tissue improved the follicular survival and development by changing the ratio of *BCL2* and *BAX* expression as apoptotic-related genes (32).

In spite of some reports regarding the positive effects of LPA on the maturation of the oocyte, based on our knowledge, there have been few studies regarding the optimal concentration of LPA on the development of isolated follicles.

Thus, in the present study, we applied LPA during 2 steps: in vitro culture of whole ovaries and of isolated follicles. The aims of this study were to: 1) determine the effective concentration of LPA to improve the growth and development of mouse isolated preantral follicles and subsequently their embryo development; 2) evaluate this effect on the isolated follicles that are derived from cryopreserved ovaries; 3) assess the incidence of cell death at the morphological and molecular levels in cultured isolated follicles in both vitrified and non-vitrified groups; and 4) analyze the expression of LPA receptor types 1-4 and developmental genes in cultured follicles at the end of the culture period.

## 2. Material and Methods 

### Animal and ovaries collection

The ovaries were obtained from 1-wk-old National Medical Research Institute (NMRI) female mice that were kept and bred under a 12 hr light/dark cycle at 22 ± 2 C and 40-50% humidity at Tarbiat Modares University animal house, Tehran, Iran. The mice (n = 20) were killed by cervical dislocation, and their ovaries were isolated and dissected free of fat and mesentery and placed into an alpha-minimal essential medium (α-MEM; Invitrogen, Paifley, UK), which was supplemented with 5% fetal bovine serum (FBS; Invitrogen, Paifley, UK), 100 IU/ml of penicillin (Invitrogen, Paifley, UK), 100 µg/ml of streptomycin (Invitrogen, Paifley, UK), 0.23 mM of sodium pyruvate (Sigma Aldrich, Dusseldorf, Germany) and 0.22 gr/ml of sodium bicarbonate (Sigma Aldrich, Dusseldorf, Germany).

### Experimental design

This experimental study was designed in 2 steps. In the first step, the collected whole ovaries were cultured in the presence of 20 µM of LPA for one wk (29). Then, their preantral follicles were mechanically isolated and cultured individually for 12 days in the presence of different concentrations of LPA (0, 10, 20, 40, 80 µM). The group with the highest survival and developmental rates of follicles and maturation rate of oocytes was selected for the second step of this study. In the second step, the whole ovaries were collected again and some of them were vitrified (33) and after one wk, in vitro culture as a control group, their preantral follicles were obtained, capsulated and cultured for 12 days in the presence or absence of LPA (the concentration of LPA was selected according to the results of the first step of the study). Thus, the groups of the study included non-vitrified LPA ${}^{-}$ , non-vitrified LPA ${}^{+}$ , vitrified LPA ${}^{-}$ and vitrified LPA ${}^{+}$ . The following evaluations were done for the cultured follicles in all studied groups: a viability test using Calcein AM staining, cell death analysis by flow cytometry using an annexin V/Pi kit, analysis of the expression of genes related to apoptosis, in vitro fertilization, and embryonic development.

### The first step of the study 

#### Ovarian culture and follicular isolation

The whole ovaries (n = 10 in each group) in the non-vitrified groups were cultured on inserts (Millicell-CM, 0.4-m pore size; Millipore Corp, Billerica, MA, USA) in 24-well plates at 37 C, 5% CO ${}_{2}$ for 7 days. Each well contained 400 µl of α-MEM culture medium containing 5% FBS, 1% insulin, transferrin, and selenium (ITS; Invitrogen, Paifley, UK), 100 mIU/ml of recombinant follicle stimulating hormone (rFSH or Gonal-f; Serono, Switzerland) and 20 µM of LPA (1-oleoyl-2-hydroxy-sn-glycerol-3-phosphate sodium salt; Avanti Polar Lipids, Instruchemie, The Netherlands). Every other day, 200 µl of the culture media in each well was refreshed. At the end of the culture period, by insulin-gauge needles, the preantral follicles (140-150 µm in diameter) were manually isolated (n = 50 in each group). The criteria for follicle isolation were 2-3 layers of granulosa cells, healthy and visible oocytes, and a thin layer of theca cells.

#### Encapsulation of follicles in the sodium alginate and 3-dimensional in vitro culture 

The isolated preantral follicles (n = 50 in each group, repeated 5 times) were encapsulated in sodium alginate using the method described previously (34). The sodium alginate solution 0.5% (w/v) was reconstituted with sterile phosphate buffer saline (PBS). The collected follicles were individually plunged into 5 µl droplets of sodium alginate; then they were put into 50 mM of CaCl ${}_{2}$ and 140 mM of NaCl for 2 min, and then washed in α-MEM media. After that, these follicles were cultured in the medium containing 0, 10, 20, 40, or 80 µM of LPA.

#### Assessment of follicular diameter 

Under an inverted microscope the morphology of the cultured follicles was observed every 48 hr. The normal follicles had a clear appearance and the degenerated follicles had dark and fragmented oocytes and granulosa cells (34). The diameter of the follicles was measured using a pre-calibrated ocular micrometer at x100 magnification under an inverted microscope (n = 45/each).

#### In vitro ovulation and induction 

After 12 days of in vitro culture of follicles, ovulation was induced by adding 1.5 IU/ml human chorionic gonadotropin hormone (Organon, Griekenweg, Netherlands) to the culture media (n = 50 in each group, with 5 repeats). Then, the released oocytes were classified as a germinal vesicle, germinal vesicle breakdown, or metaphase II (MII).

### The second step of the study

In the second step of this study, the whole ovaries were collected again and divided randomly into non-vitrified and vitrified groups (n = 15/ each).

#### Vitrification and warming procedures

The ovaries (n = 15) were vitrified and warmed based on the previously described protocol with some modifications (33). The ovaries were dehydrated with a vitrification medium (EFS40) containing 40% ethylene glycol (v/v), 30% ficoll 70 (w/v), and 1 M sucrose for 5 min at room temperature. Afterward, they were individually put on the CryoLock (Biotech, UK) with a minimum volume of the vitrification solution and were quickly plunged into liquid nitrogen and stored for 7 days. For warming, the CryoLocks were immersed into 1000 µl of 1 M sucrose solution at 37 C and were sequentially transferred in descending concentrations of sucrose (0.5 and 0.25 M) for 5 min (in each step) at room temperature. Warmed ovaries were equilibrated in an α-MEM medium supplemented with 5% FBS for one hr at 37 C in humidified 5% CO ${}_{2}$ air before tissue culture and evaluation.

#### 3-dimensional in vitro culture of isolated follicles in vitrified and non-vitrified groups 

The non-vitrified and vitrified/warmed ovaries were cultured (n = 15 in each group) for one wk, and then their preantral follicles were mechanically isolated (n = 380 follicles in each group), encapsulated, and cultured in the same way as described for the first step of the study. The encapsulated follicles were individually cultured (n = 340 follicles in each group) in an α-MEM medium supplemented with 5% FBS, 100 mIU/ml recombinant FSH, 1% ITS, 100 mg/ml penicillin, and 50 ng/ml streptomycin in the presence and absence of 40 µM of LPA (the concentration of LPA was selected according to the results of the first step of the study) under mineral oil at 37 C with 5% CO ${}_{2}$ for 12 days. These experiments were done at least 10 times. Every other day, 200 µl of the culture medium in each well was refreshed and the remaining media were stored separately at -20 ${}^{o}$ C for hormonal evaluation (34). Ovulation was induced after 12 days of in vitro culture of follicles by adding 1.5 IU/ml human chorionic gonadotropin hormone to the culture media (n = 50 in each group, with 5 repeats). Then the oocytes were evaluated and the MII oocytes were subjected to in vitro fertilization.

#### Evaluation of the ovarian follicular viability using Calcein AM staining

For evaluating the survival rate of the isolated follicles in the vitrified and non-vitrified groups, the isolated preantral follicles (n = 20 in each group, with 3 repeats) were stained with double fluorescent labeling dyes with Calcein AM and ethidium homodimer according to the Live/Dead Viability kit (Invitrogen, UK). The isolated preantral follicles were washed with PBS and then incubated in 1.6 µM of Calcein AM and 5.5 µM of ethidium homodimer for 30-45 min at 37 C in the dark. Then, follicles were reported as survived (stained green), dead (stained red), or damaged (stained partially green and red) under a fluorescent microscope.

#### In vitro fertilization and embryo culture

The sperm suspensions obtained from the cauda epididymis (7-8-wk-old male NMRI mice) were placed into a 500 ml drop of global medium (Life Global, UK) that was supplemented with 5 mg/ml bovine serum albumin (BSA) under mineral oil and were incubated at 37ºC in humidified 5% CO ${}_{2}$ air for 1.5 hr. The collected MII oocytes derived from all groups (n = 25 for each group) were inseminated with capacitated spermatozoa at a final concentration of 1 × 10^6^/ml in a global medium that was supplemented with 15 mg/ml BSA for 4-6 hr. Then, the oocytes were washed and cultured in global media supplemented with 5 mg/ml BSA under mineral oil at 37ºC and 5% CO ${}_{2}$ . Fertilization and development of embryos were evaluated up to 120 hr later.

#### Flow cytometry

The flow cytometry evaluation was performed to distinguish the intact, early, and late apoptotic and necrotic cells in the cultured follicles in the vitrified and non-vitrified groups. The samples were obtained from the ovarian follicles on day 12 of culture (n = 90 follicles in each group, with 3 repeats). The follicular cells were obtained by pipetting and then adding to 0.25% trypsin/0.04% EDTA in PBS, then they were washed with 2% BSA. The cell suspension (10^6^ cells/ml) was filtered through a 100-μm nylon mesh cell strainer and incubated for 10 min. Then it was washed with 2 ml of warm PBS 2 times and was incubated for 15 min in annexin V-fluorescein isothiocyanate and propidium iodide staining solution, in accordance with the instructions of a commercial assay kit (annexin V-FITC Apoptosis Detection Kit, Biotool, UK) and finally, the binding buffer was added. Using this technique, intact, early apoptotic, late apoptotic and necrotic cells showed no fluorescence, green, orange, and red fluorescence, respectively. The data of flow cytometry were assessed using flowJo software (Life Sciences, Ashland, Oregon).

#### RNA extraction

Total RNA was extracted from the cultured ovarian follicles in all groups at the end of 12 days of culture (n = 30 follicles in each group, with 3 repeats) using the trizol reagent, in accordance with the manufacturer's instructions (RNeasy Mini Kit; Qiagen, Germany). The ovarian follicles were homogenized with 0.5 ml of trizol reagent, then 0.1 ml of chloroform was added and the samples were centrifuged at 12,000 g for 10 min. The upper colorless aqueous phase was removed to a fresh 2 ml microtube and 700 μl of 100% isopropanol was added and centrifuged at 12,000 g for 10 min. Then 1.5 ml of 70% ethanol per 1 ml of trizol reagent was added. Using spectrophotometry (Eppendorf, Hamburg, Germany) RNA concentration was determined. The cDNA was synthesized from RNA by the cDNA kit (Thermo Fisher Scientific, UK), in accordance with the manufacturer's instructions, which was then stored at -20ºC until the real-time reverse transcription polymerase chain reaction (RT-PCR).

#### Real-time RT-PCR

The primers for LPAR1-4, *BAX*, *Bad*, *BCL ${}_{2}$ *, *P53*, *GDF9*, and *BMP15* were designed using the online software package Gen Bank database (http://www.ncbi.nlm.nih.gov) (Table I) and were assessed using the IDT Primer Quest tool - Oligo Analyzer (http://scitools.idtdna.com/analyzer/ Applications/OligoAnalyzer). For BLAST analysis, the NCBI database (http://www.ncbi.nlm.nih.gov/ tools/primer-blast/index) was used. Real-time RT-PCR was done using the QuantiTect SYBR Green RT-PCR kit (Applied Biosystem, USA) Each PCR reaction tube (20 μl) contained 2 μl of cDNA product, 2 μl each of forward and reverse primers, 6 μl of water, and 10 μl of SYBR Green. The real-time RT-PCR protocol was performed as the denaturation stage for 5 min (at 95ºC), cycling stage of denaturation for 15 sec (at 95ºC), annealing stage for 30 sec (at 58ºC), melting stage for 15 sec (at 72ºC), and a melt curve stage at 95ºC for 15 sec, 60ºC for one min, and 95ºC for 15 sec. To determine the relative quantity of the target genes, the Pfaffl method was used.

**Table 1:** List of primers used for qRT-PCR


** Accession numbers**	*Gene*	**Primer sequence**	**PCR product size (bp)**
** XM_011249935.1**	*LPAR1*	Forward: CTGCCTCTACTTCCAGCCCTG Reverse: GCTCACTGTGTTCCATTCTGTG	141
** NM_020028.3**	*LPAR2*	Forward: GACCACACTCAGCCTAGTCAAGAC Reverse: CTTACAGTCCAGGCCATCCA	106
** NM_022983.4**	*LPAR3*	Forward: CCACTTTCCCTTCTACTACCTGCT Reverse: GACGGTCAACGATTTCGACACC	115
** NM_175271.4**	*LPAR4*	Forward: GCCAGTTGCCAGTTTACACG Reverse: TGGACGCAGACGATCAGA GA	118
**NM-007527.3 **	*BAX*	Change forward: CGGCGAAATGGAGATGAACTG Reverse: GCAAAGTAGAAGAGGGCAACC	160
**NM-177410**	*Bcl2*	Forward: GGTGTTCAGATGTCGGTTCA Reverse: CGTCGTGACTTCGCAGAG	135
** NM_001285453.1**	*Bad*	Change forward: CGCTTAGAACTGGAGGGAGGA Reverse: CACTCGGCTCAAACTCTGGG	99
**NM-011640 **	*P53*	Forward: AGAGACCGCAGTACAGAAGA Reverse: GCATGGGCATCCTTTAACTC	227
**NM_008110.2 **	*Gdf9*	Forward: CAAACCCAGCAGAAGTCAC Reverse: AAGAGGCAGAGTTGTTCAGAG	164
**NM_009757 **	*BMP15*	Forward AAATGGTGAGGCTGGTAA Reverse: TGAAGTTGATGGCGGTAA	148

### Ethical considerations

These experiments were done according to the Ethical Guidelines for the Care and Use of Laboratory Animals and Protocols set by Tarbiat Modares University, Tehran, Iran. This experimental study was approved by the Ethical Committee of the Medical Faculty of Tarbiat Modares University, Tehran, Iran (Ref No: 52/8188).

### Statistical analysis

Data analysis was done using the Statistical Package for the Social Sciences software (V24; SPSS Inc., Chicago, IL, USA). Values were given as mean ± SD. The results of the survival and developmental rates of the cultured follicles and embryos, the diameter of the cultured follicles, flow cytometry analysis, and mRNA expression in all studied groups were compared with one-way ANOVA, and Tukey's HSD was used as post hoc tests. Statistical significance was considered as a p-value of less than 0.05 at the 95% confidence level.

## 3. Results

### The morphology of cultured ovaries

**Figure 1:**
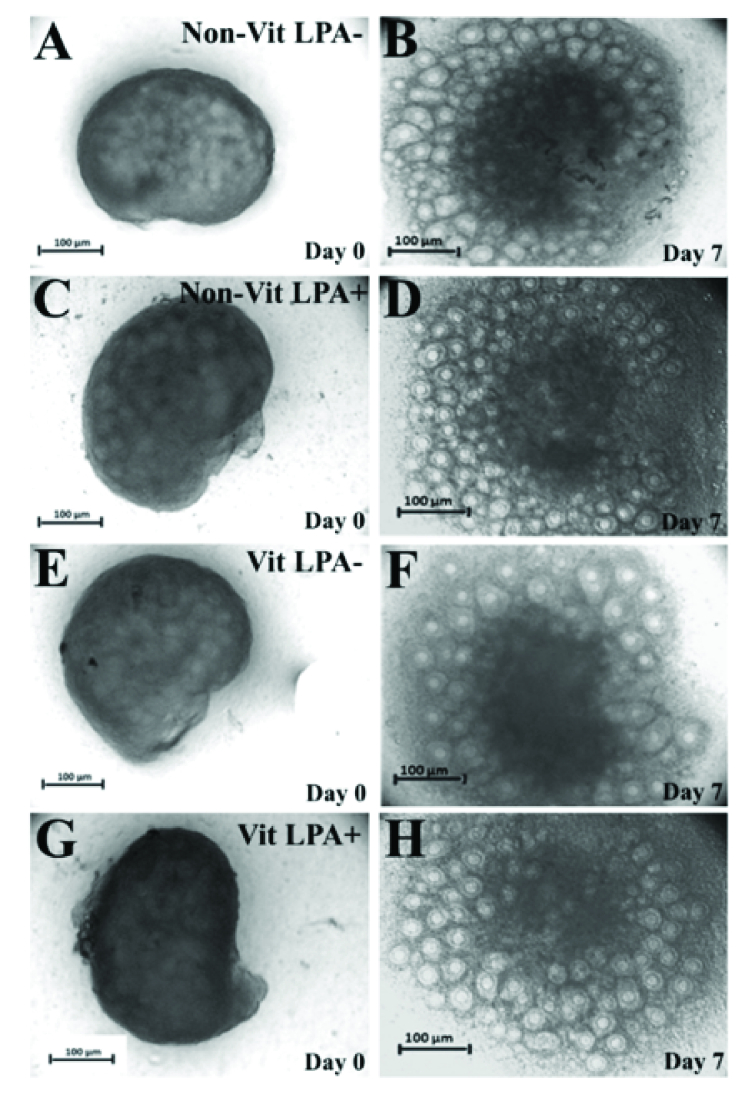
Representative photomicrographs of cultured whole ovaries in the vitrified and non-vitrified groups in the presence and absence of lysophosphatidic acid (LPA). The phase-contrast micrographs of cultured ovaries at the beginning of culture are shown in the first column (day 0) and on day 7 of the culture period in the second column. Non-vitrified in the absence of LPA (A, B), Non-vitrified in the presence of LPA (C, D), Vitrified in the absence of LPA (E, F), Vitrified in the presence of LPA (G, H)

The light microscopy of mouse whole ovaries in all studied groups are presented in figure 1. The size of the ovaries increased during the cultivation period. The follicles exhibited outgrowth in the cortical parts of the ovaries.

### The growth and developmental rates of cultured follicles in the presence of different concentrations of LPA (first step) 

The diameter and growth of the follicles cultured in different concentrations of LPA were compared within groups. The diameter of the follicles of the group with 0 µM of LPA increased from 149.56 ± 1.7 µm at the beginning of the culture period to 343.73 ± 2.4 µm at the end of the culture period. The diameter increased from 147.55 ± 1.3 µm to 358.13 ± 2.8 µm in the 5 µM LPA group, from 150.15 ± 1.4 µm to 358.25 ± 2.6 µm in the 10 µM LPA group, from 149.25 ± 1.9 µm to 368.53 ± 2.5 µm in the 20 µM LPA group, from 148.57 ± 1.8 µm to 415.85 ± 2.7 µm in the 40 µM LPA group and from 145.53 ± 1.5 µm to 372.23 ± 2.5 µm in the 80 µM LPA group (p = 0.025). In the 40 µM LPA supplemented group, the follicles had a significantly larger diameter than other groups (p = 0.025). The survival rates, antrum formation, and oocyte maturation in the isolated follicles cultured with different concentrations of LPA are summarized in table II. The highest rate of follicular survival, antrum formation, and oocyte maturation (% of MII oocytes) was achieved in the 40 µM LPA treated group (p = 0.035). Thus, the 40 µM of LPA group was selected for supplementation of culture media in the subsequent experimental groups.

### The survival rate and phase-contrast microscopy observation of isolated preantral follicles using Calcein AM staining in vitrified and non-vitrified groups

**Figure 2:**
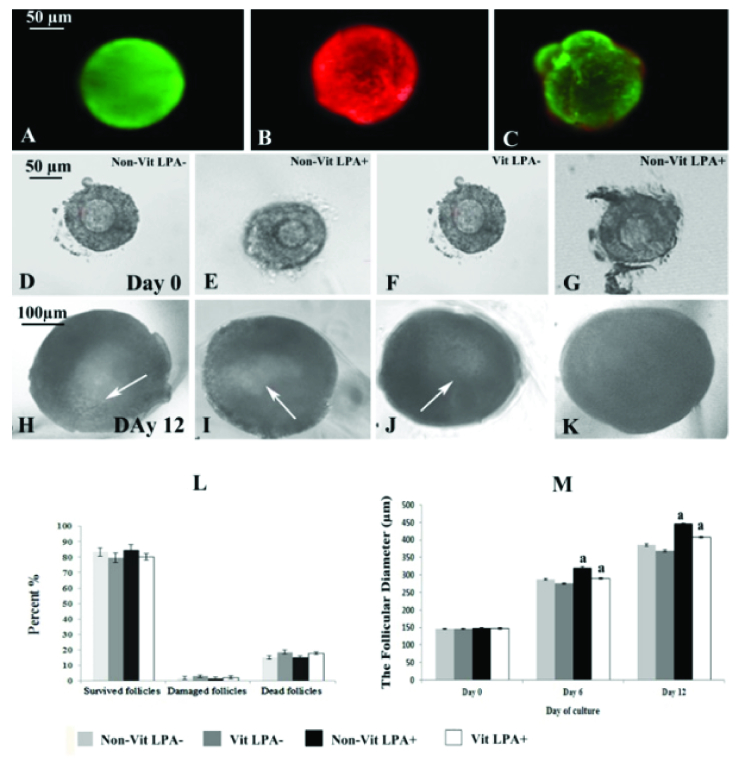
Representative fluorescence microscopy images of mechanical isolated preantral follicles from vitrified and non-vitrified cultured ovaries stained with Calcein AM are presented from A-C sections. The follicles were stained green (survived; A), red (dead; B), and damaged (green-red; C). Photomicrographs of cultured isolated follicles viewed under an inverted microscope (D-K). The phase-contrast micrographs of the cultured follicles at the beginning of culture are shown in the second row (day 0) and on day 12 of the culture period in the third row. Non-vitrified in the absence of lysophosphatidic acid (LPA) (Non-vit LPA ${}^{-}$ ; D and H), Non-vitrified in the presence of LPA (Non-vit LPA ${}^{+}$ ; E and I), Vitrified in the absence of LPA (Vit LPA ${}^{-}$ ; F and J), Vitrified in the presence of LPA (Vit LPA ${}^{+}$ ; G and K).

After the mechanical isolation of follicles from cultured ovaries, these follicles were stained by Calcein AM. The representative micrographs of survived (green), degenerated (red), and damaged (green and red) follicles are presented in figure 2 A-C. The phase-contrast morphological observations of the cultured isolated follicles in the studied groups are shown in figure 2 D-K. The size of the follicles increased during the culture period and a clear antrum formed within these follicles (white arrow). The percentages of survived, degenerated and damaged follicles were not significantly different across the studied groups (Figure 2 L).

### Diameter of cultured preantral follicles in the vitrified and non-vitrified groups

The diameter of the follicles in all groups increased during the culture period (Figure 2 M). The diameters of the follicles at the end of the culture period in the non-vitrified LPA ${}^{-}$ , non-vitrified LPA ${}^{+}$ , vitrified LPA ${}^{-}$ and vitrified LPA ${}^{+}$ were 358.63 ± 2.60, 418.15 ± 2.35, 335.31 ± 2.31, and 387.62 ± 2.74 µm, respectively. There was no significant difference in the diameter of follicles between the non-vitrified and vitrified groups, but this parameter was significantly higher in the 2 LPA supplemented groups than in their respective non-LPA treated groups (p = 0.035).

### Developmental rates of cultured preantral follicles in vitrified and non-vitrified groups

After 12 days of in vitro culture, the percentages of survived follicles, the rates of antrum formation, and the maturation rates of oocytes were compared in the vitrified and non-vitrified groups in the presence or absence of LPA and the results are presented in table III. A significantly higher percentage of survived follicles, antrum formation, and MII oocytes was observed in both of the LPA treated groups in comparison with their respective non-LPA treated samples (p = 0.035), but there was no significant difference between the vitrified and non-vitrified groups.

### Fertilization rate and embryo development in studied groups

The rat of MII oocytes that fertilized in the non-vitrified LPA ${}^{-}$ , vitrified LPA ${}^{-}$ , non-vitrified LPA ${}^{+}$ and vitrified LPA ${}^{+}$ groups were 78.57 ± 2.56%, 70.07 ± 5.01%, 82.87 ± 6.85% and 77.33 ± 5.43%, respectively (Table IV). The hatching rates of embryos in the previously listed groups were 31.81 ± 6.35, 26.31 ± 8.03, 44.82 ± 5.13, and 32.01 ± 6.31, respectively. There was no significant difference between the vitrified and non-vitrified groups in these regards, but the fertilization and hatching rates were significantly higher in the 2 LPA treated groups than in their respective non-treated groups (Table V, p = 0.028).

### Flow cytometry

The results of the flow cytometry analysis showed that there was no significant difference in the proportion of intact, apoptotic, and necrotic cells in the cultured follicles of the vitrified vs. non-vitrified groups. Moreover, in all LPA treated groups, the percentage of intact cells was significantly higher and the percentage of early and late apoptotic and necrotic cells was lower than in the non-LPA treated groups (Table V, p = 0.035).

### Real-time RT-PCR analysis 

**Figure 3:**
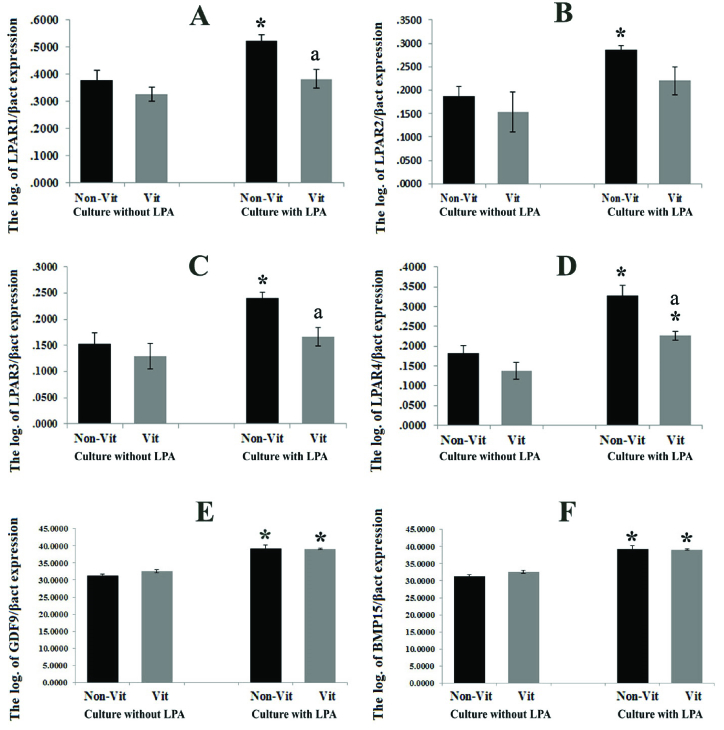
The comparison of the expression ratio of lysophosphatidic acid (LPA) receptors (LPAR1-4) and developmental genes (*GDF9* and *BMP15*) with a housekeeping gene (* β actin*) in the cultured isolated follicles in all studied groups is presented from A-F. *Significant differences with respective non-LPA supplemented group (p = 0.035). a: Significant differences with the respective non-vitrified group (p = 0.028). Non-Vit: Non-vitrified group, Vit: Vitrified group.

**Figure 4:**
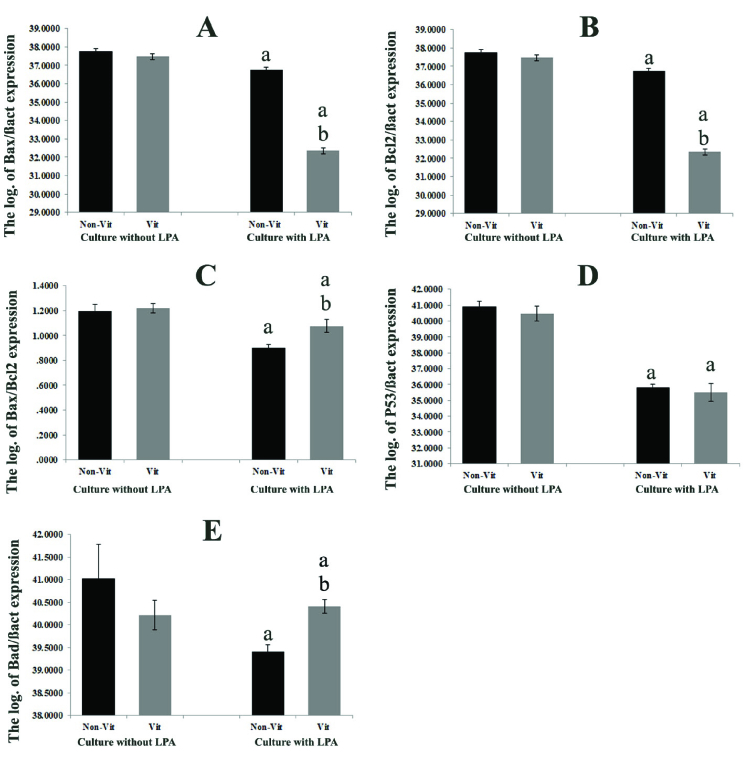
The comparison of the expression ratio of pro-apoptotic and anti-apoptotic genes (*Bax, Bcl2, P53, Bad*) with a housekeeping gene (* β actin*) in the cultured isolated follicles in the studied groups is shown in parts A-E. a: Significant differences with respective non-LPA supplemented group (p = 0.035). b: Significant differences with respective non-vitrified group (p = 0.038). Non-Vit: Non-vitrified group, Vit: Vitrified group, LPA: Lysophosphatidic acid.

The expression ratio of the LPAR1-4 genes to the *β -actin* gene in the studied groups was demonstrated and compared in figure 3 A-D. These ratios were significantly higher in all LPA treated groups in comparison with the non-treated groups (p = 0.035). In addition, this expression ratio was significantly lower in the vitrified groups than in the non-vitrified groups (p = 0.028). The mRNA levels of *GDF9* and *BMP15* in the vitrified and non-vitrified groups in the presence or absence of LPA were compared and shown in figure 3 E-F. The expression of the *GDF9* and *BMP15* genes was significantly higher in the groups that were cultured in the presence of LPA in comparison with those cultured in the absence of LPA, but there was no significant difference between the vitrified and non-vitrified groups. The expression ratio of the apoptotic-related genes including *Bax*, *Bcl2*, *P53*, and *Bad* in the non-vitrified and vitrified groups in the LPA supplemented and non-supplemented groups were compared and demonstrated in figure 4. The expression ratios of the pro-apoptotic genes *Bax* and *Bad* were significantly lower in the LPA supplemented groups in comparison with the non-LPA supplemented groups (p = 0.035). Moreover, there was a significant difference between the vitrified and non-vitrified groups in the presence of LPA (p = 0.028). In addition, there was a significantly lower ratio of *BCL2*/*BAX* expression in the vitrified group in comparison with the non-vitrified group (p = 0.028).

**Table 2:** The developmental and maturation rates of the cultured isolated follicles in the presence of different concentrations of LPA


** LPA (µM)**	**No. of survived follicles**	**No. of antrum formations**	**No. of GVs**	**No. of MIs**	**No. of MIIs**
** 0**	76 (72.39 ± 3.78)	44 (57.89 ± 3.58)	24 (31.57 ± 2.46)	29 (38.15 ± 2.54)	23 (30.26 ± 3.54)
** 5**	78 (76.47 ± 4.25)	47 (60.25 ± 3.09)	23 (29.48 ± 3.15)	32 (41.02 ± 3.62)	23 (29.48 ± 2.85)
** 10**	95 (78.51 ± 3.48)	60 (63.15 ± 2.18)	27 (28.42 ± 2.46)	39 (41.05 ± 1.56)	29 (30.52 ± 1.58)
** 20**	94 (82.45 ± 2.56)	64 (68.08 ± 2.63)	22 (23.40 ± 2.67)	40 (42.55 ± 2.83)	32 (34.04 ± 2.57)
** 40 **	98 ${}^{a}$ (89.90 ± 3.45)	77 (78.57 ± 3.54)	16 (16.32 ± 1.75)	38 (38.77 ± 2.57)	44 ${}^{a}$ (44.89 ± 2.05)
** 80 **	104 (84.55 ± 3.72)	76 (73.07 ± 3.05)	23 (22.11 ± 2.56)	45 (43.26 ± 1.78)	36 (34.61 ± 3.52)
Data in parentheses are presented as Mean% ± SD. ${}^{a}$ Significant differences with other groups in the same column (p = 0.035). The developmental rates were calculated based on the survived follicles. No: Number of follicles in each stage, LPA: Lysophosphatidic acid, GV: Germinal vesicle, MI: Metaphase I, MII: Metaphase II					

**Table 3:** The developmental and maturation rates of cultured isolated follicles in all studied groups


** Group**	**Total no. of follicles**	**No. of survived follicles**	**No. of antrum formations**	**No. of GVs**	**No. of MIs**	**No. of MIIs**
** Non-vitrified LPA ${}^{-}$ **	105	77/105 (73.33 ± 4.72)	45/77 (58.44 ± 3.65)	22/77 (28.57 ± 2.64)	31/77 (40.75 ± 3.51)	24/77 (31.16 ± 3.54)
** Vitrified LPA ${}^{-}$ **	125	90/125 (72.00 ± 3.83)	51/90 (56.66 ± 3.28)	30/90 (33.33 ± 3.76)	34/90 (37.77 ± 2.87)	26/90 (28.88 ± 3.42)
** Non-vitrified LPA ${}^{+}$ **	130	116/130 (89.23 ± 4.25) ${}^{*}$	91/116 (78.44 ± 3.28) ${}^{*}$	19/116 (16.37 ± 2.08) ${}^{*}$	45/116 (38.79 ± 3.74) ${}^{*}$	56/116 (48.27 ± 3.82) ${}^{*}$
** Vitrified LPA ${}^{+}$ **	114	92/114 (80.70 ± 3.46) ${}^{*}$	63/92 (68.47 ± 3.51) ${}^{*}$	32/92 (34.78 ± 3.12) ${}^{*}$	26/92 (28.27 ± 3.62) ${}^{*}$	34/92 (36.95 ± 2.90) ${}^{*}$
Data in parentheses are presented as Mean% ± SD. *Significant differences with respective non-treated LPA group (p = 0.035). The developmental rates were calculated based on the survived follicles. No: Number of follicles in each stage, LPA: Lysophosphatidic acid, GV: Germinal vesicle, MI: Metaphase I, MII: Metaphase II						

**Table 4:** The embryo developmental rates in all groups


** Group **	**No. of inseminated oocytes**	**No. of fertilized**	**No. of 2 cell**	**No. of the morula**	**No. of hatched**
** Non-vitrified LPA ${}^{-}$ **	28	22/28 (78.57 ± 2.56)	19/22 (67.85 ± 4.23)	10/22 (45.48 ± 4.25)	7/22 (31.81 ± 6.35)
** Vitrified LPA ${}^{-}$ **	26	19/26 (70.07 ± 5.01)	12/19 (63.15 ± 6.25)	8/19 (42.10 ± 7.51)	5/19 (26.31 ± 8.03)
** Non-vitrified LPA ${}^{+}$ **	33	29/33 (82.87 ± 6.85) ${}^{*}$	26/29 (89.56 ± 7.56) ${}^{*}$	17/29 (58.62 ± 5.26) ${}^{*}$	13/29 (44.82 ± 5.13) ${}^{*}$
** Vitrified LPA ${}^{+}$ **	30	25/30 (77.33 ± 5.43) ${}^{*}$	19/25 (76.00 ± 5.24) ${}^{*}$	12/25 (48.05 ± 1.25) ${}^{*}$	8/25 (32.01 ± 6.31) ${}^{*}$
Data in parentheses are presented as Mean% ± SD. *Significant differences with respective LPA ${}^{-}$ groups (p = 0.035). No: Number of follicles in each stage, LPA: Lysophosphatidic acid					

**Table 5:** The flow cytometry analysis of the cultured isolated follicles in the vitrified and non-vitrified groups after 12 days of culture


** Group**	**% of intact cells**	**% of early apoptotic cells**	**% of late apoptotic cells**	**% of necrotic cells**
** Non-vitrified LPA ${}^{-}$ **	71.18 ± 1.18	8.45 ± 0.74	11.41 ± 0.50	10.50 ± 2.56
** Non-vitrified LPA ${}^{+}$ **	86.42 ± 0.57 ${}^{a}$	4.46 ± 0.21 ${}^{a}$	5.04 ± 0.29 ${}^{a}$	3.94 ± 0.21 ${}^{a}$
** Vitrified LPA ${}^{-}$ **	59.15 ± 1.76 ${}^{b}$	9.01 ± 0.57	15.08 ± 0.03	16.71 ± 2.02 ${}^{b}$
** Vitrified LPA ${}^{+}$ **	70.45 ± 1.51 ${}^{ab}$	5.72 ± 0.85 ${}^{a}$	9.41 ± 1.03 ${}^{a}$	13.07 ± 1.7 ${}^{b}$
Data are presented as Mean% ± SD. ${}^{a}$ Significant differences with respective lysophosphatidic acid (LPA) ${}^{-}$ group (p = 0.035), ${}^{b}$ Significant differences with respective non-vitrified follicles (p = 0.038)				

## 4. Discussion

In the present study, we examined whether LPA supplementation of culture media could improve the mouse ovarian follicular in vitro growth and development. First, we attempted to determine the most appropriate concentration of LPA for improving the in vitro maturation condition of isolated follicles. In the present study, for the first time, we revealed that among different examined concentrations of LPA, a 40 µM concentration of LPA could enhance the development and growth of isolated follicles up to the antral stage. According to our results, the follicular growth and development gradually increased as the concentration of LPA raised to 40 µM, but these rates were lower at a concentration of 80 µM of LPA.

Results obtained from other parts of this study demonstrated that LPA with 40 µM concentration had more positive effects on mouse follicular development and also on the oocyte maturation and subsequent embryo development in comparison with the non-LPA supplemented control groups. The selected concentrations of LPA in the present and previous studies (29, 30) were similar to the level of this molecule in the follicular fluid (13). Several other studies have published similar results on the effects of LPA on the in vitro maturation of oocytes in bovine and porcine species, and in the golden hamster (23-28). These studies considered a range of applied concentrations of LPA of 10-30 µM/L and our results are in line with their observations (23-28).

Also, our results suggested that LPA, as a maturation factor, can enhance the rate of follicles and oocytes that reach the antral and MII stages, respectively. Similarly, it was shown that supplementation of culture media with 30 μM of LPA improved oocyte maturation, fertilization and blastocyst formation in mice (27). Moreover, the nuclear maturation of mouse oocytes during in vitro maturation in the presence of LPA has been demonstrated by several investigators through different mechanisms such as reducing the intra-oocyte cAMP levels, by stimulating the expression of cyclin B1, via increasing mRNA follistatin and growth differentiation factor 9 (14, 17, 23, 24, 28). LPA may indirectly promote follicular development by interacting with other cell types; however, more studies are needed to prove this suggestion.

Our molecular analysis showed that the expression of 2 follicular developmental genes (*Gdf9* and *Bmp15*) was significantly higher in the LPA treated groups than in the non-treated groups. It has been demonstrated that *Gdf9* and *Bmp15* are expressed in oocytes of all follicular stages (except primordial follicles) and these genes contribute to the regulation of female reproductive functions (22, 35).

In agreement with our study, another research showed that LPA can enhance the expression of several genes related to bovine oocyte pluripotency (*OCT4* and *SOX2*) and developmental competence (*follistatin* and *Gdf9*); they also showed that LPA reduced the expression of cysteine proteinase, an enzyme which impairs the developmental potential of bovine oocytes (24).

Our results demonstrated that 4 types of LPARs (1-4) were expressed at the mRNA level in the cultured mouse follicles. Interestingly the level of LPA receptor mRNA was higher in the LPA supplemented groups. It seems that LPA may play autocrine and paracrine roles in follicular cells and oocytes. Also, related reports have confirmed that LPARs can modulate autocrine and/or paracrine functions and promote nuclear and cytoplasmic maturation of mouse oocytes (23, 24, 36).

Our results also showed that the follicular diameter was significantly higher in all LPA treated groups than in the non-treated control groups. The increase in the size of follicles could be related to proliferation and expansion of follicular and theca cells and it may be due to the growth factor-like and hormone-like activities of LPA in these cells (13). Similarly, the proliferative effect of LPA on other cells has been demonstrated in previous studies via stimulating glucose metabolism (23, 24, 37).

Our results also showed a higher percentage of intact cells and lower percentage of late apoptotic and necrotic cells in all LPA treated groups in comparison with the non-LPA treated groups. This observation was confirmed by our molecular analysis, which indicated that the expression of the pro-apoptotic genes *Bax* and *Bad* was significantly lower and the expression of the anti-apoptotic gene *Bcl2* was higher in the LPA supplemented groups. The low ratio of *Bax/BCL2* may be a good indicator of cell survival or apoptosis. Thus, our observations revealed the anti-cell death effects of LPA during the process of in vitro growth of follicles by enhancing the anti-apoptotic gene expression profile in follicular cells (23). A similar effect of LPA on mouse and human follicular development has been shown in studies by others (31, 32). Their analyses showed that the *Bax/Bcl2 *ratio was lower in ovaries that were treated with LPA compared to the non-treated groups. They revealed that LPA, as a survival factor, enhanced follicular development in mouse and human ovaries by balancing between the anti- and pro-apoptotic genes (31, 32). Similarly, it was shown that LPA enhanced *BCL2* mRNA levels and decreased the *BAX/BCL2* ratio in blastocysts (24, 38).

Also, in confirmation of our observation, the molecular analysis showed that the developmental genes *GDF9* and *BMP15* were not changed in the vitrified group. In contrast to our previous studies, we demonstrated the adverse effects of the vitrification procedure on the follicular cell survival and development after one wk of in vitro culture of mouse whole ovaries (4, 29, 30, 39). Moreover, some alterations in the LIF receptor genes and apoptosis-related genes (except *p53*) were observed in the vitrified group that was treated with LPA. In agreement with these data, the flow cytometry analysis demonstrated an increase in necrotic cells in the vitrified groups. It seems that these alterations at the molecular levels were improved during culture and there were no adverse effects on the final development to antral stages or subsequent embryonic development.

These controversial results may have been related to ignoring the damaged follicles after mechanical isolation therefore, at the final stage of experiment the intact and normal follicles were selected and studied, so this group of follicles was not shown the negative effect of vitrification.

## 5. Conclusion

LPA can improve the survival and developmental potential of isolated follicles. Despite some alterations in the expression of apoptosis-related genes in the vitrified ovaries, LPA had positive effects on the survival and development of these follicles.

##  Conflict of Interest

The authors declare that there is no conflict of interest.
